# PD-L1 is an independent prognostic predictor in gastric cancer of Western patients

**DOI:** 10.18632/oncotarget.8169

**Published:** 2016-03-18

**Authors:** Christine Böger, Hans-Michael Behrens, Micaela Mathiak, Sandra Krüger, Holger Kalthoff, Christoph Röcken

**Affiliations:** ^1^Department of Pathology, Christian-Albrechts-University, Kiel, Germany; ^2^Department of Experimental Cancer Research, Christian-Albrechts-University, Kiel, Germany

**Keywords:** programmed death-1, predictive biomarker, immune therapy, pembrolizumab

## Abstract

Targeting the PD-1/PD-L1 immune checkpoint signaling is a novel promising treatment strategy in several tumor entities, and it is suggested that PD-L1/PD-1 expression is predictive for a PD-1/PD-L1 checkpoint inhibitor treatment response. We investigated the expression of PD-L1 and PD-1 by immunohistochemistry in a large and well characterized gastric cancer (GC) cohort of Caucasian patients, consisting of 465 GC samples and 15 corresponding liver metastases. Staining results were correlated with clinico-pathological characteristics and survival. PD-L1 expression was found in tumor cells of 140 GCs (30.1%) and 9 liver metastases (60%) respectively in immune cells of 411 GCs (88.4%) and 11 liver metastases (73.3%). PD-1 was expressed in tumor infiltrating lymphocytes in 250 GCs (53.8%) and in 11 liver metastases (73.3%). PD-L1 expression was significantly more prevalent in men, GCs of the proximal stomach, unclassified, papillary, Her2/neu-positive, Epstein-Barr-virus-positive, microsatellite instable, and *PIK3CA*-mutated GCs. A high PD-L1/PD-1 expression was associated with a significantly better patient outcome, and PD-L1 turned out to be an independent survival prognosticator. The correlation of PD-L1/PD-1 expression with distinct clinico-pathological patient characteristics may serve as a surrogate marker of PD-L1-positive GCs and may direct the use of immune checkpoint treatment strategies.

## INTRODUCTION

The genetic complexity of gastric cancer (GC) has been recently shown in an integrative genomic analysis including whole-genome sequencing. A molecular classification was proposed, which categorizes four subtypes, i.e. Epstein-Barr-virus (EBV)-positive, microsatellite instable (MSI), chromosomal instable, and genomically stable GCs [1, 2]. These current findings serve as a roadmap for patient stratification and trials of targeted therapies, and it turned out that elevated PD-L1 expression was enriched in EBV-positive and MSI GCs.

PD-L1 (B7-H1) is a 290aa type I transmembrane surface glycoprotein encoded by the CD274 gene located on chromosome 9 and expressed by several cell types of the immune system, e.g. lymphocytes and dendritic cells, but also aberrantly on the surface of epithelial cells of a wide range of solid tumors. PD-L1 is the ligand of programmed cell death 1 (PD-1), which is a member of the immunoglobulin superfamily B7 and involved in immunomodulation [3]. PD-1 is known to be expressed by activated T-cells on the germinal center of lymph follicles, but also on tumor infiltrating lymphocytes (TILs) and other immune cells [4]. The interaction of PD-1 with its ligand PD-L1 induces a suppression of T-cell receptor signaling and results in a down regulation of the immune response, which enables cancer cells to escape immune destruction [5]. Therapeutic PD-1/PD-L1 checkpoint inhibitors target the PD-1/PD-L1 immune checkpoint in order to restore the cancer cell-directed immune response [6].

Currently, more than 400 studies worldwide focus on PD-L1/PD-1 immune checkpoint signaling, including 65 studies for cancer of the gastrointestinal tract [7], and there is some evidence that PD-L1 expression is associated with response to PDL-1/PD-1 pathway inhibition [8]. Preliminary results in metastatic GC with PD-1/PD-L1 checkpoint inhibitors are highly promising, and phase III studies have recently started [9]. To date, the PD-L1/PD-1 in GC was only evaluated in small cohorts of Asian patients, but not in Caucasians, whose GCs are known to hold different gene signatures [10]. Thus, data regarding the expression and impact of PD-L1/PD-1 in GC in Western patients is urgently needed, but not yet available. In order to fill this gap of information, we systematically investigated the expression of PD-L1 and PD-1 in a large and thoroughly characterized Central European GC cohort.

## RESULTS

The clinico-pathological characteristics of our patient cohort are summarized in Table 1. 465 patients fulfilled all study criteria. Overall survival data was available in 451 (97.0%) cases, tumor specific survival data in 421 (90.5%). Mean follow-up period was 20.7 months (range 0.2 to 109.0 months).

**Table 1:** Clinico-pathological patient characteristics and their correlation with PD-L1 and PD-1 expression

	Total Vaild		PD-L1 in Tumor Cells				PD-L1 in Immune Cells				PD-1 in Immune Cells			
			p-value				p-value				p-value			
			negative		positive		negative		positive		negative		positive	
	n	(%)	n	(%)	n	(%)	n	(%)	n	(%)	n	(%)	n	(%)
**Gender**	465					0.018				0.319				0.848
Female	175	(37.6)	144	(82.3)	31	(17.7)	118	(67.4)	57	(32.6)	82	(46.9)	93	(53.1)
Male	290	(62.4)	210	(72.4)	80	(27.6)	182	(62.8)	108	(37.2)	133	(45.9)	157	(54.1)
**Age Group**	465					0.588				0.245				0.710
< 68 years	232	(49.9)	174	(75.0)	58	(25.0)	156	(67.2)	76	(32.8)	105	(45.3)	127	(54.7)
≥ 68 years	233	(50.1)	180	(77.3)	53	(22.7)	144	(61.8)	89	(38.2)	110	(47.2)	123	(52.8)
**Localization**	460					<0.001				0.248				481
Proximal	145	(31.5)	95	(65.5)	50	(34.5)	88	(60.7)	57	(39.3)	63	(43.4)	82	(56.6)
Distal	315	(68.5)	256	(81.3)	59	(18.7)	210	(66.7)	105	(33.3)	149	(47.3)	166	(52.7)
**Laurén Phenotype**	464					<0.001				0.089				0.057
Intestinal	240	(51.7)	170	(70.8)	70	(29.2)	150	(62.5)	90	(37.5)	105	(43.8)	135	(56.2)
Diffuse	145	(31.3)	136	(93.8)	9	(6.2)	101	(69.7)	44	(30.3)	78	(53.8)	67	(46.2)
Mixed	31	(6.7)	27	(87.1)	4	(12.9)	23	(74.2)	8	(25.8)	16	(51.6)	15	(48.4)
Unclassified	48	(10.3)	20	(41.7)	28	(58.3)	25	(52.1)	23	(47.9)	16	(33.3)	32	(66.7)
**Mucin Phenotype**	408					0.014				0.798				0.553
Intestinal	118	(28.9)	77	(65.3)	41	(34.7)	78	(66.1)	40	(33.9)	56	(47.5)	62	(52.5)
Gastric	64	(15.7)	51	(79.7)	13	(20.3)	40	(62.5)	24	(37.5)	31	(48.4)	33	(51.6)
Mixed	157	(38.5)	128	(81.5)	29	(18.5)	102	(65.0)	55	(35.0)	71	(45.2)	86	(54.8)
Unclassified	69	(16.9)	55	(79.7)	14	(20.3)	41	(59.4)	28	(40.6)	26	(37.7)	43	(62.3)
**T-Category**	463					0.001				0.005				0.932
T1a	11	(2.4)	11	(100.0)	0	(0.0)	10	(90.9)	1	(9.1)	6	(54.5)	5	(45.5)
T1b	44	(9.5)	35	(79.5)	9	(20.5)	30	(68.2)	14	(31.8)	20	(45.5)	24	(54.5)
T2	54	(11.7)	28	(51.9)	26	(48.1)	23	(42.6)	31	(57.4)	22	(40.7)	32	(59.3)
T3	186	(40.2)	149	(80.1)	37	(19.9)	118	(63.4)	68	(36.6)	88	(47.3)	98	(52.7)
T4a	128	(27.6)	99	(77.3)	29	(22.7)	88	(68.8)	40	(31.2)	58	(45.3)	70	(54.7)
T4b	40	(8.6)	30	(75.0)	10	(25.0)	29	(72.5)	11	(27.5)	20	(50.0)	20	(50.0)
**N-Category**	462					0.412				0.223				0.106
N0	132	(28.6)	97	(73.5)	35	(26.5)	76	(57.6)	56	(42.4)	57	(43.2)	75	(56.8)
N1	67	(14.5)	47	(70.1)	20	(29.9)	48	(71.6)	19	(28.4)	34	(50.7)	33	(49.3)
N2	81	(17.5)	63	(77.8)	18	(22.2)	54	(66.7)	27	(33.3)	46	(56.8)	35	(43.2)
N3(a/b)	182	(39.4)	144	(79.1)	38	(20.9)	119	(65.4)	63	(34.6)	76	(41.8)	106	(58.2)
**M-Category**	465					0.029				<0.001				0.641
M0	373	(80.2)	276	(74.0)	97	(26.0)	226	(60.6)	147	(39.4)	170	(45.6)	203	(54.4)
M1	92	(19.8)	78	(84.8)	14	(15.2)	74	(80.4)	18	(19.6)	45	(48.9)	47	(51.1)
**Liver Metastases**	465					1.000				0.439				0.460
no	448	(96.3)	341	(76.1)	107	(23.9)	287	(64.1)	161	(35.9)	209	(46.7)	239	(53.3)
yes	17	(3.7)	13	(76.5)	4	(23.5)	13	(76.5)	4	(23.5)	6	(35.3)	11	(64.7)
**UICC-Stage**	463					0.010				0.001				0.855
IA	44	(9.5)	37	(84.1)	7	(15.9)	31	(70.5)	13	(29.5)	20	(45.5)	24	(54.5)
IB	34	(7.3)	21	(61.8)	13	(38.2)	16	(47.1)	18	(52.9)	14	(41.2)	20	(58.8)
IIA	57	(12.3)	42	(73.7)	15	(26.3)	34	(59.6)	23	(40.4)	27	(47.4)	30	(52.6)
IIB	45	(9.7)	28	(62.2)	17	(37.8)	29	(64.4)	16	(35.6)	19	(42.2)	26	(57.8)
IIIA	52	(11.2)	35	(67.3)	17	(32.7)	30	(57.7)	22	(42.3)	29	(55.8)	23	(44.2)
IIIB	80	(17.3)	67	(83.8)	13	(16.2)	42	(52.5)	38	(47.5)	35	(43.8)	45	(56.2)
IIIC	61	(13.2)	46	(75.4)	15	(24.6)	44	(72.1)	17	(27.9)	26	(42.6)	35	(57.4)
IV	90	(19.4)	76	(84.4)	14	(15.6)	72	(80.0)	18	(20.0)	44	(48.9)	46	(51.1)
**Lymph Node Ratio**	461					0.016				0.174				0.852
< Median	227	(49.2)	161	(70.9)	66	(29.1)	139	(61.2)	88	(38.8)	106	(46.7)	121	(53.3)
≥ Median	234	(50.8)	189	(80.8)	45	(19.2)	158	(67.5)	76	(32.5)	107	(45.7)	127	(54.3)
**L-Category**	461					0.146				0.005				0.849
L0	216	(48.8)	158	(73.1)	58	(26.9)	125	(57.9)	91	(42.1)	99	(45.8)	117	(54.2)
L1	227	(51.2)	180	(79.3)	47	(20.7)	161	(70.9)	66	(29.1)	107	(47.1)	120	(52.9)
**V-Category**	442					0.595				0.113				0.069
V0	393	(88.9)	302	(76.8)	91	(23.2)	249	(63.4)	144	(36.6)	177	(45.0)	216	(55.0)
V1	49	(11.1)	36	(73.5)	13	(26.5)	37	(75.5)	12	(24.5)	29	(59.2)	20	(40.8)
**Grade**	463					0.377				0.652				0.450
G1/G2	114	(24.6)	83	(72.8)	31	(27.2)	71	(62.3)	43	(37.7)	49	(43.0)	65	(57.0)
G3/G4	349	(75.4)	269	(77.1)	80	(22.9)	227	(65.0)	122	(35.0)	165	(47.3)	184	(52.7)
**R-Status**	463					0.138				0.141				0.574
R0	401	(87.4)	301	(75.1)	100	(24.9)	254	(63.3)	147	(36.7)	183	(45.6)	218	(54.4)
R1/R2	58	(12.6)	49	(84.5)	9	(15.5)	43	(74.1)	15	(25.9)	29	(50.0)	29	(50.0)
***H. pylori*-status**	392					0.625				0.665				0.126
Negative	331	(84.4)	254	(76.7)	77	(23.3)	212	(64.0)	119	(36.0)	155	(46.8)	176	(53.2)
Positive	61	(15.6)	45	(73.8)	16	(26.2)	37	(60.7)	24	(39.3)	22	(36.1)	39	(63.9)
**EBV-Status**	451					<0.001				0.003				0.021
Negative	431	(95.6)	338	(78.4)	93	(21.6)	281	(65.2)	150	(34.8)	203	(47.1)	228	(52.9)
Positive	20	(4.4)	2	(10.0)	18	(90.0)	6	(30.0)	14	(70.0)	4	(20.0)	16	(80.0)
**MSI-Status**	450					0.001				0.282				0.606
MSS	414	(92.0)	321	(77.5)	83	(22.5)	268	(64.7)	146	(35.3)	188	(45.4)	226	(54.6)
MSI	36	(8.0)	18	(50.0)	18	(50.0)	20	(55.6)	16	(44.4)	18	(50.0)	18	(50.0)
**Her2/neu-Status**	433					0.001				0.596				0.040
Negative	396	(91.5)	307	(77.5)	89	(22.5)	253	(63.9)	143	(36.1)	188	(47.5)	208	(52.5)
Positive	37	(8.5)	19	(51.4)	18	(48.6)	22	(59.5)	15	(40.5)	11	(29.7)	26	(70.3)
**MET-Status**	454					0.528				0.704				0.274
Negative	422	(93.0)	318	(75.4)	104	(24.6)	272	(64.5)	150	(35.5)	193	(45.7)	229	(54.3)
Positive	32	(7.0)	26	(81.2)	6	(18.8)	22	(68.8)	10	(31.2)	18	(56.2)	14	(43.8)
***KRAS*-Genotype**	457					0.031				0.595				0.612
Wildtype	441	(96.5)	339	(76.9)	102	(23.1)	287	(65.1)	154	(34.9)	207	(46.9)	234	(53.1)
Mutated	16	(3.5)	8	(50.0)	8	(50.0)	9	(56.2)	7	(43.8)	6	(37.5)	10	(62.5)
***PIK3CA*-Genotype**	457					0.006				0.004				0.012
Wildtype	436	(95.4)	337	(77.3)	99	(22.7)	289	(66.3)	147	(33.7)	209	(47.9)	227	(52.1)
Mutated	21	(4.6)	10	(47.6)	11	(52.4)	7	(33.3)	14	(66.7)	4	(19.0)	17	(81.0)
***RHOA*-Genotype**	396					0.203				0.397				0.789
Wildtype	382	(96.5)	287	(75.1)	95	(24.9)	244	(63.9)	138	(36.1)	173	(45.3)	209	(54.7)
Mutated	14	(3.5)	13	(92.9)	1	(7.1)	7	(50.0)	7	(50.0)	7	(50.0)	7	(50.0)
*GNAS1*-Genotype	440					0.086				0.865				1.000
Negative	399	(90.7)	305	(76.4)	94	(23.6)	251	(62.9)	148	(37.1)	183	(45.9)	216	(54.1)
Positive	41	(9.3)	26	(63.4)	15	(36.6)	27	(65.9)	14	(34.1)	19	(46.3)	22	(53.7)
**Overall Survival [Months]**						0.028(1)				<0.001(1)				0.185(1)
Total / Events / Censored	451 / 353 / 98		344 / 276 / 68		107 / 77 / 30		291 / 245 / 46		160 / 108 / 52		209 / 165 / 44		242 / 188 / 54	
Median Survival	15.0±1.1		14.6±1.1		18.8±5.8		12.6±1.1		22.6±3.9		14.2±1.3		16.5±2.1	
95% C.I.	12.8-17.2		12.5-16.7		7.4-30.2		10.5-14.7		15.0-30.2		11.6-16.7		12.4-20.6	
**Tumor Specific Survival [Months]**						0.018(2)				<0.001(2)				0.050(1),(2)
Total / Events / Censored	421 / 288 / 133		319 / 228 / 91		102 / 60 / 42		269 / 202 / 67		152 / 86 / 66		196 / 145 / 51		225 / 143 / 82	
Median Survival	16.7±1.5		16.0±1.3		25.0±8.8		13.5±1.1		29.5±5.0		15.5±1.6		18.0±2.9	
95% C.I.	13.8-19.6		13.5-18.5		7.8-42.2		11.4-15.7		19.7-39.3		12.3-18.6		12.4-23.6	

EBV denotes Epstein-Barr virus, MSI microsatellite instability

(1) insignificance after multiple testing.

(2) Log-rank test was applied to survival analyses; all other Fisher's exact test

### PD-L1 and PD-1 expression

#### PD-L1

**Figure 1:**
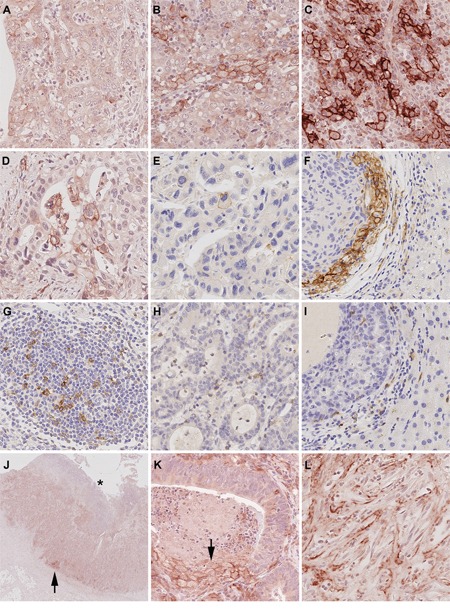
PD-L1 and PD-1 expression in gastric cancer and liver metastases The entire gastric cancer (GC) cohort was screened and three representative cases were selected as reference slides for PD-L1 1+ **A.** 2+ **B.** and 3+ **C.** PD-L1 expression was observed in primary GCs **D.** and its corresponding liver metastases **E.** Microsatellite instable GCs often showed an increased PD-L1 expression at the interface between tumor and non-neoplastic tissue, which could also be observed in GC liver metastases **F.** PD-1 positive lymphocytes were present in the majority of intratumoral lymph follicles **G.** PD-1 expression in diffuse tumor infiltrating lymphocytes (TILs; **H.**) correlated significantly with patient survival and was also observed in liver metastases **I.** PD-L1 positive tumor cells were close to the tumor surface (distance <2.5 mm, equivalent of an average biopsy forceps) and generally attainable by endoscopic biopsy 66.9%. However, in 39 cases (33.1%), PD-L1 positive tumor cells were localized only in the tumor center (**J.**; arrow head; the asterisk marks the mucosal surface) or near the invasion front. Necrotic tumor cells were found to show strong membranous PD-L1-expression (**K.**; arrow). PD-L1 positive stroma cells **(L.)** Original magnifications 2-fold **J.** and 400-fold (**A-I.**, **K-L.**).

**Figure 2:**
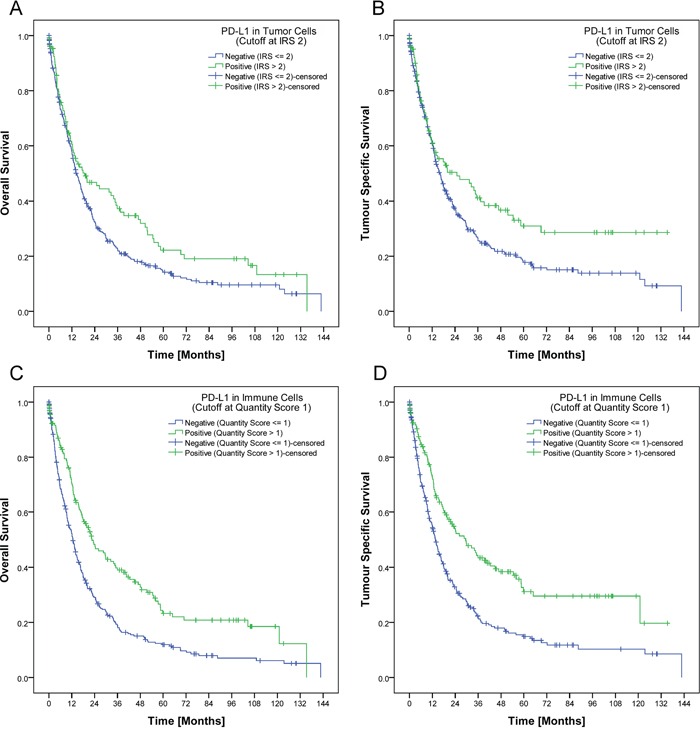
Development of a biomarker score In our study, a tumor was regarded as PD-L1 positive if it had a least 2% tumor cells with an at least moderate membranous positivity IRS (>2) respectively at least 10% PD-L1 positive immune cells. Applying this cut-off, patients with PD-L1 positive tumor cells had a significant better overall (p=0.028; **A.**) and tumor specific survival (p=0.018; **B.**). The same applied for patients with PD-L1 positive immune cells. PD-L1 expression in immune cells was significantly associated with a better overall and tumor specific survival (p<0.001 each; **C.**, **D.**).

PD-L1 expression was observed in tumor, stromal and immune cells, but not in non-neoplastic gastric epithelium. 140 of 465 cases (30.1%) showed a membranous PD-L1 expression in tumor cells. The percentage of stained tumor cells ranged from 0 to 80% (median 0%), the staining intensity ranged from 0 to 3 (median 0; Figure 1A-1C; Table 1). Only a minority of cases showed different staining intensities within the same tumor. Although we aimed for the HistoScore, we recognized that the overall percentage of PD-L1-positive tumor cells was low (in 90% of the cases <10% immunopositive tumor cells; Table 2) and assessment of different percentages of three different staining intensities was indiscernible and impractical. Therefore, the application of the HistoScore was discarded and a simplified IRS was applied [11]. The tumor cell IRS ranged from 0 to 7 (median 0). Dichotomized by an IRS of 2, 111 cases (23.9%) were classified as positive and 354 cases (76.1%) as negative (Table 2; Figure 2). Additionally, intratumoral necrosis with a membranous PD-L1 staining of necrotic tumor cells was found in 72 of 465 cases (15.5%; Figure 1K). Statistical analysis was carried out only for staining of vital tumor cells.

**Table 2:** Distribution of PD-L1 expression in tumor cells for the entire cohort, GCs with distinct phenotypic and genotypic characteristics and Laurén subgroups

	Total		PD-L1 Type								Laurén phenotype								Histology of MSI cases					
Membraneous PD-L1 staining of tumor			EBV positive		MSI		Papillary type		Other		Intestinal		Diffuse		Mixed		Unclassified		No peculiarities		Single peculiar features		Highly peculiar	
	n	(%)	n	(%)	n	(%)	n	(%)	n	(%)	n	(%)	n	(%)	n	(%)	n	(%)	n	(%)	n	(%)	n	(%)
**PD-L1 Quantity Score**																								
0 (0%)	325	(69.9)	1	(5.0)	16	(44.4)	7	(23.3)	282	(78.3)	149	(62.1)	133	(91.7)	27	(87.1)	15	(31.2)	10	(76.9)	5	(71.4)	0	(0)
1 (<= 1%)	80	(17.2)	3	(15.0)	5	(13.9)	14	(46.7)	58	(16.1)	57	(23.8)	7	(4.8)	2	(6.5)	14	(29.2)	1	(7.7)	0	(0)	2	(16.7)
2 (2% - 10%)	47	(10.1)	9	(45.0)	12	(33.3)	9	(30.0)	17	(4.7)	29	(12.1)	4	(2.8)	2	(6.5)	12	(25.0)	2	(15.4)	2	(28.6)	7	(58.3)
3 (11% - 50%)	10	(2.2)	5	(25.0)	2	(5.6)	0	(0)	3	(0.8)	3	(1.2)	1	(0.7)	0	(0)	6	(12.5)	0	(0)	0	(0)	2	(16.7)
4 (> 50%)	3	(0.6)	2	(10.0)	1	(2.8)	0	(0)	0	(0)	2	(0.8)	0	(0)	0	(0)	1	(2.1)	0	(0)	0	(0)	1	(8.3)
Total	465	(100)	20	(100)	36	(100)	30	(100)	360	(100)	240	(100)	145	(100)	31	(100)	48	(100)	13	(100)	7	(100)	12	(100)
Total / Missing / p-Value (1)			446 / 19 / <0.001								464 / 1 / <0.001								32 / 433 / <0.001					
**PD-L1 Intensity Score**																								
0 (negative)	325	(69.9)	1	(5.0)	16	(44.4)	7	(23.3)	282	(78.3)	149	(62.1)	133	(91.7)	27	(87.1)	15	(31.2)	10	(76.9)	5	(71.4)	0	(0)
1 (weak)	36	(7.7)	2	(10.0)	4	(11.1)	9	(30.0)	21	(5.8)	27	(11.2)	4	(2.8)	0	(0)	5	(10.4)	2	(15.4)	0	(0)	1	(8.3)
2 (medium)	88	(18.9)	12	(60.0)	14	(38.9)	14	(46.7)	48	(13.3)	58	(24.2)	6	(4.1)	3	(9.7)	21	(43.8)	1	(7.7)	2	(28.6)	9	(75.0)
3 (strong)	16	(3.4)	5	(25.0)	2	(5.6)	0	(0)	9	(2.5)	6	(2.5)	2	(1.4)	1	(3.2)	7	(14.6)	0	(0)	0	(0)	2	(16.7)
Total	465	(100)	20	(100)	36	(100)	30	(100)	360	(100)	240	(100)	145	(100)	31	(100)	48	(100)	13	(100)	7	(100)	12	(100)
Total / Missing / p-Value (1)			446 / 19 / <0.001								464 / 1 / <0.001								32 / 433 / <0.001					
**PD-L1 IRS (Sum Score)**(3)																								
0	325	(69.9)	1	(5.0)	16	(44.4)	7	(23.3)	282	(78.3)	149	(62.1)	133	(91.7)	27	(87.1)	15	(31.2)	10	(76.9)	5	(71.4)	0	(0)
2	29	(6.2)	1	(5.0)	2	(5.6)	7	(23.3)	19	(5.3)	21	(8.8)	3	(2.1)	0	(0)	5	(10.4)	1	(7.7)	0	(0)	0	(0)
3	56	(12.0)	3	(15.0)	5	(13.9)	9	(30.0)	39	(10.8)	40	(16.7)	5	(3.4)	2	(6.5)	9	(18.8)	1	(7.7)	0	(0)	3	(25.0)
4	36	(7.7)	7	(35.0)	10	(27.8)	7	(23.3)	12	(3.3)	23	(9.6)	2	(1.4)	1	(3.2)	10	(20.8)	1	(7.7)	2	(28.6)	6	(50.0)
5	11	(2.4)	4	(20.0)	1	(2.8)	0	(0)	6	(1.7)	5	(2.1)	1	(0.7)	1	(3.2)	4	(8.3)	0	(0)	0	(0)	1	(8.3)
6	5	(1.1)	2	(10.0)	1	(2.8)	0	(0)	2	(0.6)	0	(0)	1	(0.7)	0	(0)	4	(8.3)	0	(0)	0	(0)	1	(8.3)
7	3	(0.6)	2	(10.0)	1	(2.8)	0	(0)	0	(0)	2	(0.8)	0	(0)	0	(0)	1	(2.1)	0	(0)	0	(0)	1	(8.3)
Total	465	(100)	20	(100)	36	(100)	30	(100)	360	(100)	240	(100)	145	(100)	31	(100)	48	(100)	13	(100)	7	(100)	12	(100)
Total / Missing / p-Value (1)			446 / 19 / <0.001								464 / 1 / <0.001								32 / 433 / 0.001					
**PD-L1 Status**																								
negative (IRS <= 2)	354	(76.1)	2	(10.0)	18	(50.0)	14	(46.7)	301	(83.6)	170	(70.8)	136	(93.8)	27	(87.1)	20	(41.7)	11	(84.6)	5	(71.4)	0	(0)
positive (IRS > 2)	111	(23.9)	18	(90.0)	18	(50.0)	16	(53.3)	59	(16.4)	70	(29.2)	9	(6.2)	4	(12.9)	28	(58.3)	2	(15.4)	2	(28.6)	12	(100)
Total	465	(100)	20	(100)	36	(100)	30	(100)	360	(100)	240	(100)	145	(100)	31	(100)	48	(100)	13	(100)	7	(100)	12	(100)
Total / Missing / p-Value (1)			446 / 19 / <0.001								464 / 1 / <0.001								32 / 433 / <0.001					

(1) Fisher's exact test

(2) Log-rank test

(3) Sum of Quantitiy Score and Intensity Score; n.c.: cannot be calculated; n.a.: no data available

PD-L1 expression in immune cells was found in 411 of 465 cases (88.4%) also serving as a positive control for the negatively rated tumor cell cases. The percentage of stained immune cells ranged from 0 to 70% (median 5%), the intensity ranged from 0 to 3 (median 1). As the majority of cases with PD-L1 positive immune cells showed a weak staining intensity (302 of 411 cases, 73.5%), the intensity of the immunostaining was neglected for further statistical analyses, and only the percentage of PD-L1 positive immune cells was considered. All cases were graded into four groups as 0 (negative), 1 (1-9% positive), 2 (10-20% positive), and 3 (>20% positive). For further statistical analyses, group 0 and 1 were summarized and classified as “PD-L1 negative in immune cells” (300 of 465 cases, 64.5%), and group 2 and 3 were classified “PD-L1 positive in immune cells” (165 of 465 cases, 35.5%).

The PD-L1 expression in stroma cells was mainly very limited to single cells, hence only the presence or absence was evaluated; PD-L1 positive stroma cells were observed in 107 of 465 cases (23.0%; Figure 1L).

#### PD-L1 expression patterns

EBV-positive GCs and MSI-carcinomas of other origin (e.g. colorectal) are known to be associated with PD-L1 overexpression [1, 14, 15]. Next we correlated the PD-L1/PD-1 staining results with the previously assessed EBV- and MSI-status and the Laurén- and WHO-phenotype (Table 1 & 2). PD-L1 expression in tumor cells was observed significantly more often, more intense and more extensive in EBV-positive, MSI, papillary type, and unclassified GCs (p<0.001 each; Table 2). Especially MSI-GCs with peculiar histologic MSI features [10] often showed a high PD-L1 expression in tumor cells (Table 2).

Moreover, four different PD-L1 expression patterns were observed: (1) EBV-positive GCs often showed a heterogeneous, “patchy” expression pattern with a striking accumulation of PD-L1 positive tumor cells around larger blood vessels. (2) MSI-GCs were mainly PD-L1-positive at the interface between neoplastic and non-neoplastic tissue, especially in areas of “pushing borders”. (3) Papillary type GCs often showed PD-L1 positivity within the fibrovascular connective tissue cores and the intratumoral necrosis. (4) Other cases mainly showed no distinct PD-L1 distribution pattern and were classified as “patternless” (Supplementary File 1).

#### PD-1

PD-1 expression was observed neither in tumor nor stroma cells. PD-1 positive diffusely distributed TILs were present in 250 of 465 cases (53.8%). As the amount of PD-1 positive TILs was mainly restricted to single cells, a quantitative analysis was neglected. Apart from a cumulative appearance of PD-1 positive immune cells in intratumoral lymphocyte aggregates/lymph follicles (413 of 465 cases; 88.8%; Figure 1G), no distinct distribution pattern was observed. PD-1 expression in TILs was significantly correlated with PD-L1 expression in tumor, stroma and immune cells (p<0.001 each; Figure 1H).

### Clinico-pathological correlation

#### PD-L1

PD-L1 expression in tumor cells correlated significantly with gender, tumor localization, Laurén and mucin phenotype, UICC-stage, lymph node ratio, EBV-, MSI-, Her2/neu- and *PIK3CA*-status. PD-L1 expression in immune cells correlated significantly with T-, M- and L-category, UICC-stage, EBV-, and *PIK3CA*-status (Table 1). Interestingly, the percentage of PD-L1-positive tumor and immune cells increased from pT1a to pT2 and decreased thereafter. No correlation was found between the PD-L1 expression in stroma cells and clinico-pathological patient characteristics (data not shown).

#### PD-1

There was no significant correlation between the PD-1 expression in TILs and any clinico-pathological patient characteristic after Simes' multiple testing procedure (Table 1).

### Prognostic significance

#### Univariate survival analysis

**Figure 3:**
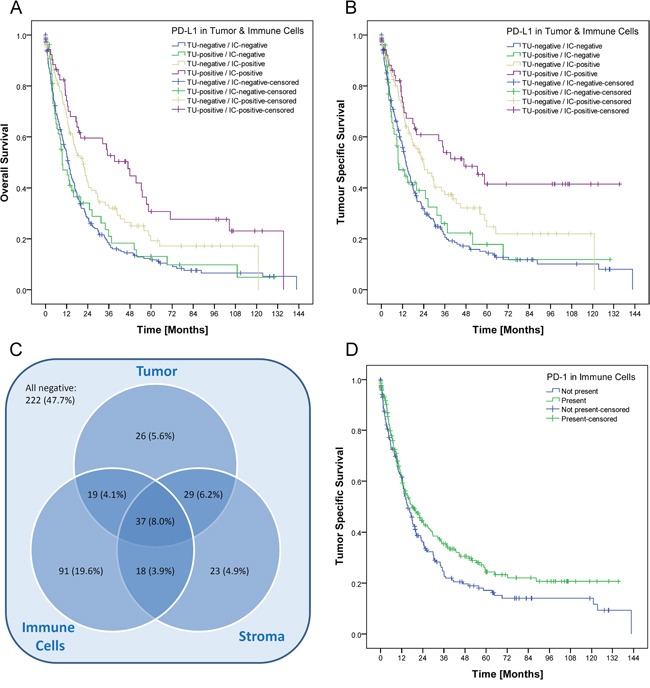
Prognostic significance and intersection of PD-L1 expression in tumor, immune and stromal cells The best overall **A.** and tumor specific survival **B.** was observed for those patients with a high PD-L1 expression in tumor cells and in immune cells (p<0.001 each). After grouping and dichotomization, 243 of 465 cases (52.3%) were classified as “PD-L1 positive” for at least one tumor component (tumor cells, stroma cells and/or immune cells; **C.**). Expression in tumor cells, stroma cells and immune cells were significantly correlated with each other (p<0.001 each). Patients with PD-1 positive TILs showed a better tumor specific survival, which was not significant after Simes' multiple testing procedure (p=0.050; **D.**).

Patient prognosis significantly depended on several clinico-pathological parameters as well as on the PD-L1 expression in tumor cells and immune cells. Patients with a high PD-L1 expression in tumor cells (IRS>2) or immune cells (group 2 and 3) showed a significant better overall and tumor specific survival (Figure 2A-2D; Table 1). This effect could not be reproduced if the IRS-cut off for tumor cells respectively the cut-off for immune cells was marked down to 0 (Supplementary File 2). The best overall and tumor specific survival was observed for those patients with a high PD-L1 expression in tumor cells and in immune cells (Figure 3A–3B). There was no significant correlation between PD-L1 expression in stroma cells and patient survival (data not shown). In general, PD-L1-status correlated highly significantly between tumor and immune cells, tumor and stroma cells, and immune and stroma cells (p<0.001 each; Figure 3C).

Patients with PD-1 positive TILs showed a better tumor specific survival, which was not significant after Simes' multiple testing procedure (Table 1; Figure 3D).

#### Multivariate survival analysis (Cox regression)

A Cox regression was carried out on all parameters which had a p<0.1 in univariate survival analysis, i.e. Laurén-phenotype, T-, N-, M-, L-, V-, and R-category, UICC-stage, lymph node ratio, tumor grade, MSI-, MET-, PD-L1- (tumor and immune cells), and PD-1-status (immune cells). Five parameters remained in the Cox model after running the backward LR method with p_in_=0.05 and p_out_=0.10. These were UICC-stage, lymph node ratio, R-status, MET-status and PD-L1 expression in immune cells (Table 3).

**Table 3:** Univariate and multivariate survival analysis

	Overall Survival						Tumor Specific Survival					
	Univariate Cox Regression			Multivariate Cox Regression*			Univariate Cox Regression			Multivariate Cox Regression*		
	HR	95% C.I.	p-Value	HR	95% C.I.	p-Value	HR	95% C.I.	p-Value	HR	95% C.I.	p-Value
UICC-Stage			<0.001			<0.001			<0.001			<0.001
IB vs. IA	1.279	0.614-2.667	0.511	1.569	0.685-3.593	0.286	2.101	0.782-5.648	0.141	4.182	1.102-15.864	0.035
IIA vs. IA	2.197	1.221-3.953	0.009	2.669	1.352-5.267	0.005	3.632	1.572-8.394	0.003	7.578	2.273-25.263	0.001
IIB vs. IA	2.503	1.357-4.614	0.003	2.564	1.268-5.184	0.009	4.741	2.046-10.985	<0.001	7.914	2.371-26.413	0.001
IIIA vs. IA	3.874	2.170-6.915	<0.001	3.249	1.613-6.544	0.001	6.925	3.060-15.674	<0.001	9.145	2.738-30.543	<0.001
IIIB vs. IA	4.888	2.816-8.486	<0.001	3.543	1.766-7.109	<0.001	9.769	4.435-21.516	<0.001	11.367	3.411-37.882	<0.001
IIIC vs. IA	7.918	4.474-14.014	<0.001	5.217	2.520-10.801	<0.001	15.184	6.786-33.971	<0.001	15.248	4.488-51.807	<0.001
IV vs. IA	9.398	5.416-16.310	<0.001	5.885	2.900-11.942	<0.001	17.520	7.945-38.630	<0.001	17.529	5.226-58.790	<0.001
LNR>Median (0.22)	3.044	2.423-3.825	<0.001	1.698	1.225-2.353	0.001	3.475	2.691-4.487	<0.001	1.684	1.163-2.438	0.006
R1/R2 vs. R0	3.701	2.734-5.012	<0.001	2.243	1.593-3.158	<0.001	4.220	3.057-5.826	<0.001	2.544	1.768-3.661	<0.001
MSI vs. MSS	0.538	0.342-0.847	0.007				0.384	0.215-0.686	0.001	0.541	0.291-1.005	0.052
MET positive	2.826	1.905-4.193	<0.001	2.324	1.500-3.600	<0.001	2.841	1.839-4.387	<0.001	2.385	1.476-3.853	<0.001
PD-L1 in tumor cells	0.753	0.584-0.971	0.029				0.710	0.533-0.944	0.018			
PD-L1 in immune cells	0.590	0.470-0.741	<0.001	0.594	0.461-0.765	<0.001	0.555	0.431-0.716	<0.001	0.599	0.451-0.795	<0.001
PD-1 in immune cells							0.793	0.629-1.001	0.051			

* Input variables: Laurén-phenotype, T-, N-, M-, L-, V- and R-category, UICC-stage, lymph node ratio, tumor grade, MSI-, MET-, PD-L1- (tumor and immune cells) and PD-1-status (immune cells).

LNR denotes lymph node ratio, MSI microsatellite instable and MSS microsatellite stable

### Expression in liver metastases

Tissue specimens from liver metastases were available in 15 cases. Nine metastases (60%) showed a membranous PD-L1 expression of tumor cells, which was mainly limited to single cells and ranged from 0.1 to 3% respectively from 1+ to 2+ (Figure 1D-1F). Dichotomized by an IRS >2, 5 cases were classified as “PD-L1 positive” in tumor cells. Twelve of 15 cases (80%) showed concordant staining results for the primary and its corresponding metastasis (κ=0.471; p=0.032).

PD-L1 expression in immune cells was found in 11 metastases (73.3%) and ranged from 1 to 10% (Figure 1I). Dichotomized by the 10% cut-off, one case (6.7%) was classified as PD-L1 positive in immune cells. Twelve of 15 cases (80%) showed concordant staining results for the GC and its corresponding metastasis (κ=0.328; p=0.086).

PD-1 positive TILs were present in 11 metastases (73.3%). The expression in GCs and their corresponding metastases was concordant in 12 cases (80%; κ=0.526; p=0.039; Supplementary File 3).

### Accessibility for biopsy diagnostics

In a palliative setting, only tumor biopsies may be available for testing of a predictive biomarker. Thus we explored the spatial distribution of PD-L1 positive tumor cells in the primary tumor. In 22 of 140 cases (15.7%) with a membranous PD-L1 expression in tumor cells, the tumor surface could not be evaluated. In the remaining 118 cases, PD-L1 positive tumor cells were close to the mucosal surface and generally accessible by biopsy in 79 (66.9%) cases. In 39 cases (33.1%), PD-L1 positive tumor cells were localized only in the tumor center or near the invasion front (Figure 1J).

## DISCUSSION

### PD-L1 and PD-1 are biologically and prognostically relevant in GC

The present study is the first evaluation of PD-L1 and PD-1 expression in a large and well-characterized Caucasian cohort of GC. PD-L1 and PD-1 were expressed in a substantial amount of GCs either in tumor and immune cells or immune cells only. PD-L1 expression was found to be an independent survival prognosticator and correlated with distinct clinico-pathological patient characteristics.

Up to now, only five studies of smaller Asian patient cohorts investigated the PD-L1/PD-1 expression in GC [12–16]. PD-L1 was found in 43 to 63% of the Asian patients and, different from our cohort, showed a negative impact on patient survival. However, Asian and non-Asian GCs exhibit distinct tumor immunity signatures related to T-cell function [10], which may also affect the correlation of PD-L1/PD-1 expression with patient survival. In other tumor entities like malignant melanoma or lung cancer, it is proven that PD-L1 positive tumors show significant higher response rates to anti-PD-1/PD-L1 therapy. Thus, treatment independent effects on patient survival have to be considered in PD-L1/PD-1-positive GCs and have to be distinguished from therapeutic effects. Still, PD-L1/PD-1 expression might serve as a predictive biomarker for PD-1/PD-L1 checkpoint inhibitor treatment, which raises several questions: What needs to be considered in the development of a PD-L1 biomarker score? Is it possible to reliably evaluate the PDL1/PD-1 expression by a biopsy? Which clinico-pathological patient characteristics may serve as a surrogate marker for PD-L1/PD-1-expression, in order to minimize the risk of sampling errors, e.g. in a palliative setting?

### PD-L1/PD-1 as putative predictive biomarkers

Accurate interpretation of immunohistochemical stains is crucial for the establishment of a valid, histology-based predictive biomarker. Although we aimed for the HistoScore, we recognized that the overall percentage of PD-L1-positive tumor cells was low and the assessment of different percentages of three different staining intensities was indiscernible and impractical. Applying our simplified IRS, we next used patient survival as surrogate marker for biological relevance. Interestingly, while dichotomization into completely negative and any positive staining of tumor cells did not show a significant correlation with patient survival (Supplementary File 2), dichotomization into PD-L1 IRS ≤1 and >2 correlated significantly with patient survival (Figure 2). Thus, minimal expression of PD-L1 may have no effect on tumor biology. Similarly, only the presence of PD-1 positive diffusely distributed TILs, and not intratumoral lymph follicles, correlated significantly with patient survival. Based on our findings we suggest that a putative, PD-L1/PD-1-based immunohistochemical predictive biomarker score should not only explore tumor cells but also intratumoral immune cells.

### PD-L1 evaluation in biopsies carries the risk of sampling errors

A significant number of patients is diagnosed at an advanced, inoperable stage [17]. Only biopsies might be available in these patients for biomarker testing. Our study shows that intratumoral heterogeneity also applies to PD-L1 expression in GC: In 39 cases (33.1%) PD-L1 positive tumor cells may not be sampled by a superficial biopsy. Accordingly, one third of PD-L1-positive GCs might carry the risk of a non-representative, i.e. false-negative, test result. This also may influence results of clinical trials exploring the value of PD-L1 as predictive biomarker in biopsy specimens.

In patients with a metastatic and/or unresectable GC, biopsy specimens might be obtained from the liver metastases, rather than the primary tumor, and biomarker expression between the primary GC and the liver metastasis might also show divergent test results. For Her2/neu, a discordance rate between 9 and 16% is known [18]. In our study, PD-L1 and PD-1 were expressed in a significant portion of liver metastases, and concordant staining with the primary was found in 80%. Yet, PD-L1/PD-1 positive GCs had PD-L1/PD-1 negative liver metastases, and vice versa. The presence of a PD-L1 and PD-1 expression in liver metastases alone is an interesting finding, which might be relevant for clinical trials regarding immune checkpoint inhibitor treatment of metastatic disease. In this context, it was interesting to note that PD-L1-expression was high in pT2-tumors and lower in pT3 and pT4-tumours. Thus, PD-L1-expression may change during tumor progression, and analysis of liver metastases in PD-L1-negative primary GCs may be eligible.

### The association of PD-L1 expression with distinct phenotypes and genotypes might be helpful in the patient selection for a targeted PD-L1/PD-1 testing and therapy

In view of non-representative tissue sampling issues, we were next interested to test the hypothesis whether other clinico-pathological patient characteristics may serve as surrogate markers for PD-L1/PD1-expression. Correlation of PD-L1/PD-1 expression with various clinico-pathological patient characteristics demonstrated that PD-L1 is enriched in men, intestinal, unclassified or papillary type GC of the proximal stomach, EBV-positive, and MSI GCs. The latter findings confirm independently previous observations [1, 19, 20]. EBV-positive GCs show a wide spectrum of histological characteristics but are often characterized by a marked lymphoid infiltration. MSI GCs predominantly consist of highly pleomorphic tumor cells, which are surrounded by a dense inflammatory stroma with little or no desmoplastic stroma reaction and often show pushing margins [21]. Thus, case selection may not only rely on immunohistochemical staining patterns, but also on distinct clinico-pathological characteristics thereby reducing the risk of sampling errors of biopsy specimens.

### The finding that PD-L1 expression is associated with the Her2/neu-status might open novel treatment strategies

The simultaneous application of separate monoclonal antibodies (mAbs) or multi-specific mAbs is still in its infancies but might offer new treatment strategies for complex diseases [22]. Stagg et al. demonstrated the enhanced efficacy of anti–ErbB-2 with anti–PD-1 mAbin transgenic mice and thereby raised the possibility that anti–PD-1 mAb therapy could be used to capitalize on the immune-mediated effects of trastuzumab [23]. We found out that PD-L1-positive GCs hold a Her2/neu-overexpression in nearly 50%, which raises hopes that similar treatment strategies might be applicable in humans. In addition, PD-L1-expression correlated significantly with *PIK3CA*-mutational status and combination of PD-L1/PD-1-targeted therapies with mTOR-inhibitors merit consideration in GC.

### Conclusion

PD-L1 expression in GC of Western patients correlates significantly with overall and tumor specific survival as well as distinct clinico-pathological patient characteristics. Our findings might help to develop valid predictive test algorithms, which do not rely only on immunostaining patterns and may also help to explore novel combination therapies, e.g. trastuzumab or everolimus and PD-1/PD-L1 checkpoint inhibitors.

## MATERIALS AND METHODS

### Ethics

All procedures followed were in accordance with the ethical standards of the responsible committee on human experimentation (institutional and national) and with the Helsinki Declaration of 1964 and later versions. Informed consent or substitute for it was obtained from all patients for being included in the study. Ethical approval was obtained from the local ethical review board (D 453/10).

### Study population

From the archive of the Institute of Pathology, University Hospital Kiel, we sought Caucasian patients who had undergone either total or partial gastrectomy for adenocarcinoma of the stomach or esophago-gastric junction between 1997 and 2009. The following patient characteristics were retrieved: type of surgery, age at diagnosis, gender, tumor size, tumor localization, tumor type, tumor grade, depth of invasion, residual tumor status, number of lymph nodes resected, and number of lymph nodes with metastases. Patients were included if an adenocarcinoma of the stomach or esophago-gastric junction was histologically confirmed. Exclusion criteria were defined as 1) histology identified a tumor type other than adenocarcinoma, and 2) patients had undergone a perioperative chemo- or radiotherapy. Each resected specimen had undergone gross sectioning and histological examination by trained and board certified surgical pathologists. Date of patient death was obtained from the *Epidemiological Cancer Registry* of the state of Schleswig-Holstein, Germany. Follow-up data of those patients who were still alive were retrieved from hospital records and general practitioners. All patient data were pseudonymized prior to study inclusion.

### Histology

Tissue specimens were fixed in formalin and embedded in paraffin (FFPE). Deparaffinized sections were stained with hematoxylin and eosin. Histological re-examination of primary tissue sections was carried out for all cases to assure if inclusion criteria were met. Tumors were classified according to the Laurén classification [24] and re-examined by two surgical pathologists (CB, CR). pTNM-stage of all study patients was determined according to the 7^th^ edition of the UICC guidelines [25].

### Immunohistochemistry of PD-L1 and PD-1

Immunostaining was carried out manually, using a rabbit monoclonal anti-PD-L1 antibody (1:75, E1L3N, CellSignaling, Danvers, United States of America). Tissue sections were pretreated in citrate buffer for antigen retrieval and incubated with hydrogen peroxide block and Ultra V Block (both Thermo Scientific, Braunschweig, Germany) to avoid unspecific reactions. For visualization the ImmPRESS-HRP-Universal–Antibody Polymer and the NovaRED substrate kit (both VectorLabs, Peterborough, United Kingdom) were applied. Counterstaining was done with hematoxylin (Dr. K. Hollborn & Söhne GmbH & Co KG; Leipzig, Germany). Immunohistochemical PD-L1 stainings of liver metastases and PD-1 stainings of GCs and liver metastases were carried out with a Bondmax automated slide staining system (Leica Biosystems, Wetzlar, Germany), using the Polymer Refine Detection Kit (Menarini Diagnostics, Berlin, Germany) and the anti-PD-L1 antibody described above respectively a mouse monoclonal anti-PD-1-antibody (clone MRQ-22, Cell Marque, Rocklin, United States of America). Germinal centers of lymph follicles served as internal positive control for both antibodies.

### Evaluation of immunostaining

Intensity and percentage of stained cells was evaluated separately for tumor, stroma and immune cells by two pathologists (CB and CR). For the evaluation of the PD-L1 expression in tumor cells, only the membranous staining was evaluated and the following immunoreactivity scoring system (IRS) was applied: Category A rated the percentage of immunoreactive cells and was graded as 0 (negative), 1 (≤1% positive), 2 (2 to 10% positive), 3 (11-50%) and 4 (>50%). Category B documented the intensity of immunostaining as 0 (no immunostaining), 1 (weak), 2 (moderate), or 3 (strong). The addition of category A and B resulted in an IRS ranging from 0 to 7. The evaluation of PD-L1 in stroma cells was rated as present or absent. For the evaluation of the PD-L1 expression in immune cells (lymphocytes, dendritic cells, macrophages), only the percentage of positive cells was considered, and cases were graded as 0 (negative), 1 (1-5% positive), 2 (6-20%) and 3 (>20%). The immunostaining of PD-1 in immune cells was rated separately for tumor infiltrating lymphocytes (TILs) and intratumoral lymph follicles as present or absent.

### Immunohistochemistry, detection and assessment of phenotypic and genotypic characteristics

The mucin phenotype, Helicobacter pylori-, Her2/neu-, MET-, EBV-, MSI-status as well as the *KRAS-*, *PIK3CA-*, *RHOA-*, and *GNAS*-genotype was assessed as previously described [11, 21, 26–29].

### Study design

Whole tissue sections from GCs and their liver metastases were stained with antibodies directed against PD-L1 and PD-1. The staining results were correlated with clinico-pathological and survival data.

### Statistical analysis

Statistical analyses were done using SPSS 20.0 (IBM Corporation, New York, USA). For the evaluation of PD-L1 in tumor cells and immune cells, marker expressions were first examined as raw score values and then dichotomized into positive and negative. Cross tabulations of clinical data and marker expressions were tested for independence using Fisher's exact test. The correlation between the PD-L1 and PD-1 expression in GCs and corresponding metastases was calculated by using Cohen's kappa. A kappa value of 0.20 was considered to be poor, of 0.21–0.40 to be fair, of 0.41–0.60 to be moderate, of 0.61–0.80 to be good, and of 0.81–1.00 to be very good. Median overall and tumour specific survival were calculated using the Kaplan-Meier method. Log-rank test was used to determine significance of differences between survival curves. Hazard ratios of variables were calculated by univariate Cox regression and those having p-values up to 0.1 were included in a multivariate Cox regression, combined with iterative backward LR method to identify independent prognostic variables. P-values up to 0.5 were considered as statistically significant. False discovery rate of correlations between clinical variables and biomarkers was controlled by applying the explorative Simes (Benjamini-Hochberg) procedure group-wise for each biomarker [30]. P-values are given unadjusted, but those having lost significance under the explorative Simes procedure are marked appropriately.

## SUPPLEMENTARY FILES




