# Supplementary material for: PD-L1 is an independent prognostic predictor in gastric cancer of Western patients
Source: Oncotarget. 2016 Mar 18;7(17):24269–83. doi: 10.18632/oncotarget.8169 (PMC5029700; doi:10.18632/oncotarget.8169)
Supplement: Supplementary file 1 [file oncotarget-07-24269-s001.pdf]

SUPPLEMENTARY FILES

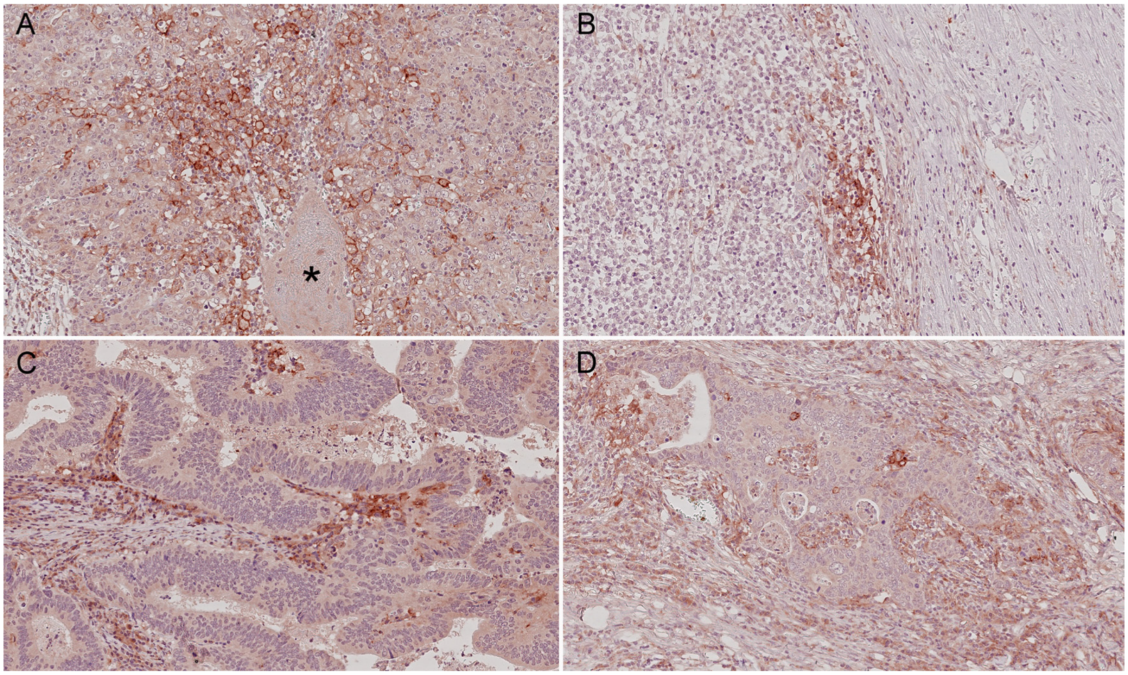

Supplementary File S1: Four different PD-L1 expression patterns were observed. Epstein-Barr-virus-positive gastric cancer often showed a heterogeneous, “patchy” expression pattern with a striking accumulation of PD-L1 positive tumor cells around larger blood vessels (**A.** asterisk marks a blood vessel). Microsatellite instable gastric cancers were mainly PD-L1-positive at the interface between neoplastic and non-neoplastic tissue, especially in areas of “pushing borders” **B.** Papillary type gastric cancers often showed PD-L1 positivity within the fibrovascular connective tissue cores **C.** Other cases mainly showed no distinct PD-L1 distribution pattern and were classified as “patternless” **D.** Original magnifications 200-fold.

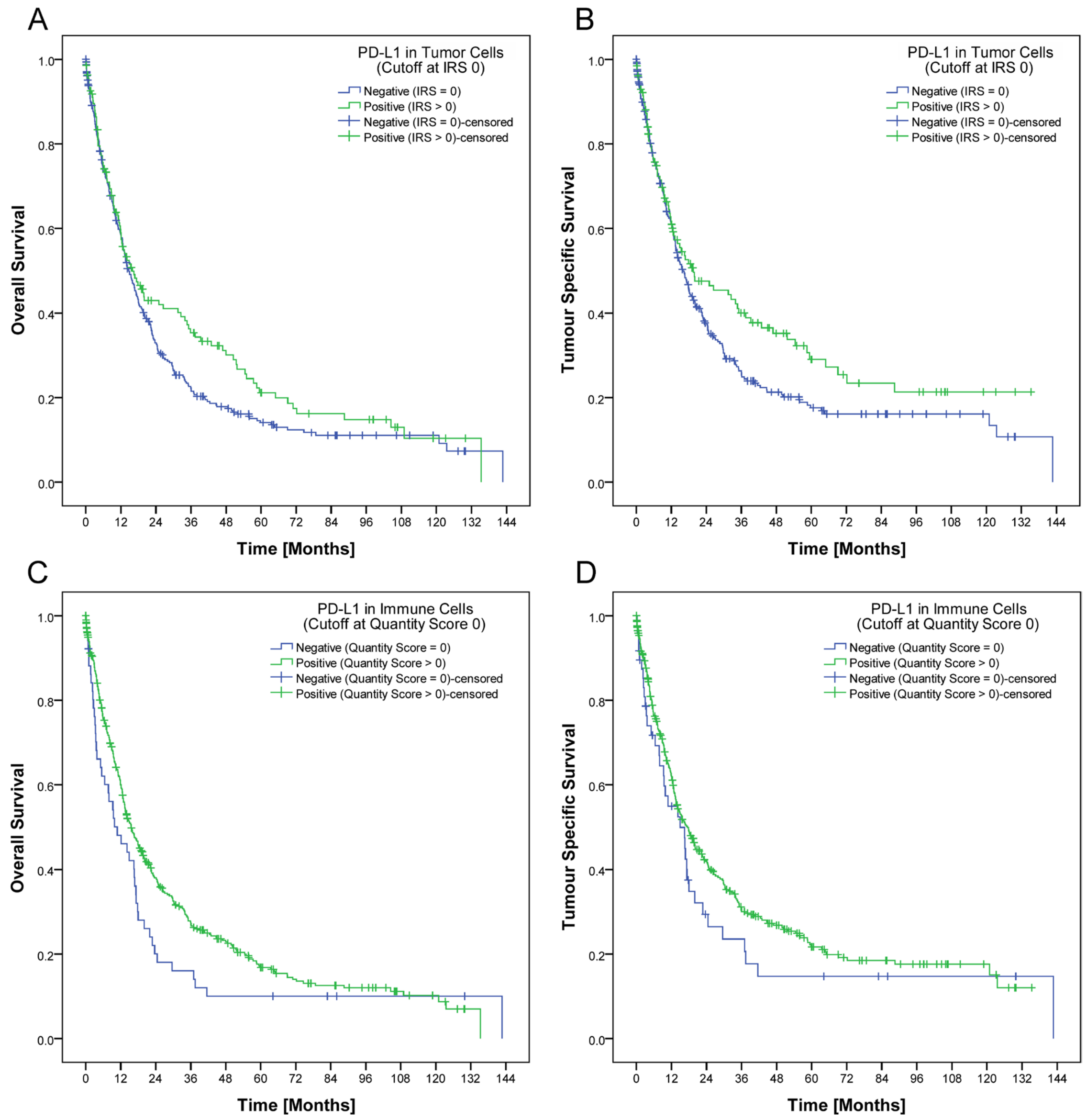

Supplementary File S2: Kaplan-Meier curves for PD-L1 expression in tumor and immune cells with a cut-off ">0": p=0.116 **A.**, p=0.064 **B.**, p=0.056 **C.**, p=0.200 **D.**

Supplementary File S3: PD-L1/PD-1 expression in gastric carcinomas and corresponding liver metastases

See Supplementary File 1
