# Supplementary material for: PD-L1 is an independent prognostic predictor in gastric cancer of Western patients
Source: Oncotarget. 2016 Mar 18;7(17):24269–83. doi: 10.18632/oncotarget.8169 (PMC5029700; doi:10.18632/oncotarget.8169)
Supplement: Supplementary file 2 [file oncotarget-07-24269-s002.docx]

**Supplemental File 3:** PD-L1/PD-1 expression in gastric carcinomas and corresponding liver metastases

| **Case number** | **Gender** | **Age at diagnosis** | **Tumor localization** | **Laurén phenotype** | **T-category** | **N-category** | **Tumor grade** | **PD-L1 in**  **tumor cells*** | | **PD-L1 in**  **immune cells**** | | **PD-1 in immune cells** | | **Distinct feature**  **(EBV, MSI, papillary )** |
| --- | --- | --- | --- | --- | --- | --- | --- | --- | --- | --- | --- | --- | --- | --- |
|  |  |  |  |  |  |  |  | **Primary tumor** | **Liver metastasis** | **Primary tumor** | **Liver metastasis** | **Primary tumor** | **Liver metastasis** |  |
| 1 | M | 74 | Distal | Unclassified | T3 | N3a | G3 | - | - | - | - | + | + | no |
| 2 | F | 61 | Proximal | Intestinal | T3 | N3a | G3 | - | - | - | - | - | - | no |
| 3 | F | 73 | Distal | Intestinal | T3 | N3a | G3 | - | - | - | - | + | + | no |
| 4 | M | 71 | Distal | Intestinal | T2 | N0 | G3 | - | - | + | - | - | + | MSI |
| 5 | F | 78 | Proximal | Intestinal | T4a | N3b | G3 | - | - | + | - | + | - | Papillary |
| 6 | F | 82 | Proximal | Intestinal | T4b | N3a | G2 | + | + | - | - | - | + | no |
| 7 | M | 66 | Distal | Intestinal | T4a | N3b | G3 | + | + | - | - | + | + | no |
| 8 | M | 73 | Proximal | Intestinal | T3 | N3b | G3 | - | + | - | - | + | + | Papillary |
| 9 | M | 69 | Proximal | Intestinal | T4b | N3b | G2 | - | + | + | - | + | + | Papillary |
| 10 | M | 78 | Distal | Intestinal | T3 | N3b | G3 | - | + | + | + | + | + | EBV |
| 11 | F | 65 | Distal | Intestinal | T4a | N3b | G3 | - | - | - | - | + | + | no |
| 12 | F | 66 | Proximal | Intestinal | T3 | N3b | G3 | - | - |  | - | + | + | no |
| 13 | M | 68 | Proximal | Mixed | T4a | N3a | G3 | - | - | - | - | - | - | no |
| 14 | M | 73 | Distal | Unclassified | T3 | N3b | G3 | - | - | - | - | - | - | no |
| 15 | F | 64 | Distal | Intestinal | T3 | N3a | G3 | - | - | - | - | + | + | no |
|  | | | | | | | | **κ=0.471;p=0.032** | | **κ=0.328;p=0.086** | | **κ=0.526;p=0.039** | |  |

*=dichotomized at IRS >2

**=dichotomized at 10%
